# Vitamin C and common cold-induced asthma: a systematic review and statistical analysis

**DOI:** 10.1186/1710-1492-9-46

**Published:** 2013-11-26

**Authors:** Harri Hemilä

**Affiliations:** 1 Department of Public Health, POB 41, University of Helsinki, Mannerheimintie 172, FIN-00014 Helsinki, Finland

**Keywords:** Anti-asthmatic agents, Ascorbic acid, Asthma, Bronchial provocation tests, Bronchoconstriction, Common cold, Forced expiratory flow rates, Histamine, Rhinovirus, Upper respiratory tract infections

## Abstract

**Background:**

Asthma exacerbations are often induced by the common cold, which, in turn, can be alleviated by vitamin C.

**Objective:**

To investigate whether vitamin C administration influences common cold-induced asthma.

**Methods:**

Systematic review and statistical analysis of the identified trials. Medline, Scopus and Cochrane Central were searched for studies that give information on the effects of vitamin C on common cold-induced asthma. All clinically relevant outcomes related to asthma were included in this review. The estimates of vitamin C effect and their confidence intervals [CI] were calculated for the included studies.

**Results:**

Three studies that were relevant for examining the role of vitamin C on common cold-induced asthma were identified. The three studies had a total of 79 participants. Two studies were randomized double-blind placebo-controlled trials. A study in Nigeria on asthmatics whose asthma attacks were precipitated by respiratory infections found that 1 g/day vitamin C decreased the occurrence of asthma attacks by 78% (95% CI: 19% to 94%). A cross-over study in former East-Germany on patients who had infection-related asthma found that 5 g/day vitamin C decreased the proportion of participants who had bronchial hypersensitivity to histamine by 52 percentage points (95% CI: 25 to 71). The third study did not use a placebo. Administration of a single dose of 1 gram of vitamin C to Italian non-asthmatic common cold patients increased the provocative concentration of histamine (PC_20_) 3.2-fold (95% CI: 2.0 to 5.1), but the vitamin C effect was significantly less when the same participants did not suffer from the common cold.

**Conclusions:**

The three reviewed studies differed substantially in their methods, settings and outcomes. Each of them found benefits from the administration of vitamin C; either against asthma attacks or against bronchial hypersensitivity, the latter of which is a characteristic of asthma. Given the evidence suggesting that vitamin C alleviates common cold symptoms and the findings of this systematic review, it may be reasonable for asthmatic patients to test vitamin C on an individual basis, if they have exacerbations of asthma caused by respiratory infections. More research on the role of vitamin C on common cold-induced asthma is needed.

## Introduction

Moses Maimonides, a 12th-century physician, wrote about asthma: “I conclude that this disorder starts with a common cold, especially in the rainy season…” [1]. Consistent with this statement, recent prospective studies have detected respiratory viruses in up to 80% of asthma exacerbations of children and adults [1-5]. The severity of the cold in asthmatics within its first two days predicted the subsequent severity of the asthma exacerbation [6]. The common cold may lead to a transient bronchial hypersensitivity, which is one characteristic of asthma [7-12]. Hypothetically, preventing or alleviating common cold symptoms might reduce the incidence and severity of asthma exacerbations caused by respiratory viruses.

Vitamin C was identified in the early 1900s, in the search for the etiology of scurvy [13]. After its identification, there was much interest in the effects of vitamin C on diseases unrelated to scurvy, but its role against other diseases is still undetermined. In placebo-controlled trials 1 g/day or more of vitamin C shortened the duration of colds in adults by 8% and in children by 18% [14-17]. The common cold studies did not examine the effect of vitamin C on pulmonary functions, but two trials found a greater effect on lower respiratory symptoms than on upper respiratory symptoms. Elwood et al. found that vitamin C significantly decreased the incidence of “chest colds” (−18%; cough or other chest symptoms) but not of “simple colds” (+1%; runny nose or sneezing) [18,19]. Anderson et al. found that vitamin C significantly decreased the incidence of “throat colds” (−21%) but not of “nose colds” (−2%) [18,20]. Furthermore, vitamin C prevented pneumonia in three controlled trials with participants under special conditions [17,21].

The use of vitamin C for treating asthma dates back to the 1940s. A few physicians reported that vitamin C seemed beneficial for some of their asthma patients, but other physicians found no such improvements in their asthma patients [22,23]. A recent meta-analysis of three randomized trials on vitamin C and exercise-induced bronchoconstriction found that vitamin C halved the post-exercise decline of forced expiratory volume in 1 second (FEV_1_), which indicates that vitamin C has effects on some phenotypes of asthma [24].

This study was motivated by the findings that asthma exacerbations are often induced by the common cold, which in turn is alleviated by vitamin C. The objective of this systematic review was to summarize the evidence on the possible role of vitamin C administration on common cold-induced asthma.

## Methods

### Types of studies

Intervention studies, randomised and non-randomised, and placebo-controlled and non-placebo-controlled, that give information on the effect of vitamin C on common cold-induced asthma and/or bronchial hypersensitivity were included in this systematic review.

### Types of participants

Studies on children and adults of either sex at any age were eligible.

### Types of interventions

The interventions considered were the oral or intravenous administration of vitamin C (ascorbic acid or its salts) as a single dose or as multiple doses for a period.

### Outcomes

All clinically relevant outcomes related to asthma such as the number of asthma exacerbations, the severity of asthma, airway hypersensitivity and pulmonary functions were included in this review.

### Literature searches

Medline (OVID) was searched using terms: (exp Ascorbic acid/ or ascorb*.mp) and (exp Asthma/ or asthma*.mp or bronch*.mp) and (exp Common cold/ or exp Respiratory Tract Infections/ or respiratory infect*.mp). Similar searches were carried out via Scopus and the Cochrane Central Register of Controlled Trials. No language restrictions were used. The databases were searched from their inception to September 2013. Studies that fulfilled the selection criteria were included. The reference lists of the identified studies and relevant review articles were screened for additional references. Finally, a cited article search of the Web of Science database was carried out to search for papers that cited the trials identified in the primary search. See Additional file 1 for the flow diagram of the literature search.

### Selection of studies and data extraction

Three studies that were relevant to the investigation of the role of vitamin C on common cold-induced asthma were identified (Tables 1 and 2). The data of the three included trials were extracted and analyzed by this author (see below and Additional files 2 and 3). Dr. Bucca was contacted for the original data, but she no longer retained those data. Dr. Bucca reported the histamine PC_20_ values of their study as figures in two separate reports [25,26]. In the current study, the individual-level values were measured from one of the figures [26]; see Additional file 2 for the data extraction. The reconstructed data set has the same means and gives the same F-statistics as Bucca et al. reported; see Additional files 2 and 3.

**Table 1:** Characteristics of the included trials

**Study**	**Item**	**Description**
Anah et al. 1980 [27]	Participants	41 asthmatic subjects attending an asthma clinic in Nigeria. All had had asthma for at least 4 yrs. The participants had histories of increased asthma attacks during the rainy season. In all cases their attacks were precipitated by respiratory infections, which started with a sore throat and a dry cough. The trial was conducted during the rainy season. Patients with bronchitis were excluded. 22 M, 19 F; age 15 to 46 y (mean 27 y); 22 vit C 19 placebo.
	Duration	14 wk
	Intervention	1 g/d vit C or a placebo for 14 wk.
	Outcome	Frequency of asthma attacks. “Severe attacks” indicate those that needed emergency attendance at the hospital; “moderate attacks” those that necessitated the use of inhalers more frequently, and “mild attacks” those that caused some increase in wheezing and breathlessness.
	Notes	See calculations in Additional files 2 and 3.
Schertling et al. 1990 [30,31]	Participants	29 Participants with a diagnosis of infection-related asthma in former East-Germany. Patients with acute and serious purulent infections were excluded. 18 M, 11 F; age 18 to 60 y.
	Duration	Total duration 5 wk, composed of 2 periods of 2 wk intervention and a 1 wk washout between them.
	Intervention	5 g/d vit C or placebo for 1 wk before the histamine sensitivity test in the middle of the 2 wk intervention. Washout 1 wk between the 2-wk intervention phases.
	Outcomes	1) Sensitivity to histamine: positive result indicates that exposure to <1 μmol histamine increased respiratory tract resistance by 50%. 2) Asthma symptom score, 3) PEF
	Notes	See calculations in Additional file 2. The histamine sensitivity data are reported for 23 participants. There is no description for the missing data.
Bucca et al. 1989 [25,26]	Participants	9 members of hospital staff in Italy with a negative history of asthma and atopy. All suffered from the common cold with cough on the first vit C test day, and all had recovered on the second vit C test day 6 wk later. 5 M, 4 F; age 18 to 48 y (mean 29 y).
	Duration	Two study days separated by 6 wk.
	Intervention	Single dose 2 g of vit C.
	Outcome	PC_20_ was measured at baseline and 1 h after vit C administration on both study days.
	Notes	See calculations in Additional files 2 and 3.

Abbreviations:

PC_20_: Concentration of histamine needed for a 20% FEV_1_ decrease.

PEF: Peak Expiratory Flow.

**Table 2:** Methodological characteristics of the included trials

**Study**	**Domain of interest**	**Description**
Anah et al. 1980 [27]	Design	Parallel-group trial.
	Randomization	Reported as a randomized trial, but the method of randomization was not described.
	Allocation concealment	Not described, but double-blinding implies that allocation must have been concealed.
	Blinding of participants and personnel	Reported as double-blind, which implies that participants and personnel were blind; however, the persons who were blind are not explicitly described.
	Blinding of outcome assessment	Reported as double-blind, which implies that outcome assessment was blind; however, the persons who were blind are not explicitly described.
	Drop-outs	No description of drop-outs.
Schertling et al. 1990 [30,31]	Design	Cross-over trial.
	Randomization	Reported as a randomized trial, but the method of randomization was not described.
	Allocation concealment	Not described, but double-blinding implies that allocation must have been concealed.
	Blinding of participants and personnel	Reported as double-blind, which implies that participants and personnel were blind; however, the persons who were blind are not explicitly described.
	Blinding of outcome assessment	Reported as double-blind, which implies that outcome assessment was blind; however, the persons who were blind are not explicitly described.
	Drop-outs	Total number of participants was 29, but histamine sensitivity is reported for 23 participants. The reasons for the 6 missing participants are not given.
Bucca et al. 1989 [25,26]	Design	Self-controlled trial. Two series of histamine challenge tests were done before and after vit C. The first series was carried out when the participants suffered from the common cold, and the second series was carried out 6 wk later after the participants had recovered. On both study days, vit C was administered after the baseline histamine challenge test and the second histamine challenge test was carried out 1 h later. No placebo.
	Randomization	Not a randomized trial.
	Allocation concealment	Not applicable.
	Blinding of participants and personnel	Not blinded.
	Blinding of outcome assessment	Not blinded.
	Drop-outs	One participant out of 10 was excluded from the statistical analysis because she had whooping cough and not the common cold.

### Statistical analysis

In 1980, Anah et al. reported the cumulative incidence of asthma attacks during the trial [27], which gives a rate ratio (RR) = 0.22 (95% confidence interval [CI]: 0.09-0.47) using the “poisson.test” program of the R-package [28]. However, Anah and colleagues did not publish the individual level data or standard deviations (SD) for the distribution of asthma attacks and therefore the variance per mean ratio could not be calculated (it is 1.0 for the Poisson distribution). Nevertheless, they published partial descriptions of the asthma attack distributions that were used to generate more realistic over-dispersed Poisson-type distributions for the treatment groups. The exact distribution of the severe and moderate asthma attacks in the vitamin C group could be inferred and thus the RR for severe and moderate attacks involves fewer imputations than the RR for all asthma attacks. The RRs and their 95% CIs were then calculated by using the “glm.nb” program of the R package, which fits the negative binomial regression model [28,29]. These conservative RR estimates are shown as the findings of the Anah study (Table 3) see Additional files 2 and 3 for the calculations.

**Table 3:** Findings of the included trials

**Study**	**Outcome**	**P (2-tail) for the difference**	**Estimate of vitamin C effect (95% CI)**	**Notes**
Anah et al. 1980 [27]	Incidence of all asthma attacks:	0.019	RR = 0.22 (0.06 to 0.81)	All asthma attacks: 9/22 and 35/19 (attacks/persons) in vit C and placebo groups, respectively. See Additional files 2 and 3 for the calculations.
	Incidence of severe and moderate asthma attacks:	0.003	RR = 0.11 (0.02 to 0.48)	Severe and moderate asthma attacks: 3/22 and 23/19 (attacks/persons) in vit C and placebo groups, respectively. See Additional files 2 and 3 for the calculations.
Schertling et al. 1990 [30,31]	Proportion of participants who were sensitive to histamine:	0.0005	52 percentage points decrease (25 to 71)	The P-value was calculated from the discordant observations: 12 were sensitive to histamine in the placebo phase but not in the vit C phase; 0 were sensitive to histamine in the vit C phase but not in the placebo phase. See Additional file 2 for the calculations.
	Asthma symptom score:	0.12	Placebo: 0.72, Vit C: 0.65	Scale 0 to 3; 0 indicates no symptoms. The P-value was calculated by Schertling et al. [30,31].
	PEF:	0.12	Placebo: 400 L/min, Vit C: 409 L/min	The P-value was calculated by Schertling et al. [30,31].
Bucca et al. 1989 [25,26]	Histamine PC_20_:	0.0003	3.2 fold increase in PC_20,_ (2.0 to 5.1 fold)	Vit C increased histamine PC_20_ geometric mean from 7.8 to 25.1 mg/ml. See Additional files 2 and 3 for the calculations.
	Interaction between the vitamin C effect and the common cold:	0.003		Interaction test for the vit C effect on PC_20_ (before/after vit C) and the presence of the common cold (yes/no). See Additional file 2 for the calculations.

Abbreviations:

CI: Confidence Interval.

PC_20_: Concentration of histamine needed for a 20% FEV_1_ decrease.

PEF: Peak Expiratory Flow.

RR: Rate Ratio.

In 1990, Schertling et al. reported the numbers of participants who were sensitive to histamine on the vitamin C and the placebo phases of a cross-over study [30,31]. Bronchial hypersensitivity was defined as a cumulative dose of <1 μmol histamine that caused a 50% increase in respiratory tract resistance. In the current study, the *P*-value for the difference in the proportions of participants with bronchial hypersensitivity to histamine between the vitamin C and the placebo phases was calculated from the discordant observations using the binomial distribution. The 95% CI for the difference in the proportions was calculated using the Agresti-Caffo method [32]. See Additional file 2 for the calculations. Schertling and colleagues did not report the distribution for asthma symptom scores or PEF values, but they reported the Wilcoxon-test *P*-values for the vitamin C and the placebo phase differences [30,31], which are shown in Table 3.

In 1989, Bucca et al. reported the histamine provocation concentrations that caused a 20% decline in FEV_1_ level (PC_20_) at baseline and at 1 hour after vitamin C administration for two study days that were separated by a 6 week interval [25,26]. On the first vitamin C test day, the participants suffered from the common cold, and 6 weeks later, on the second vitamin C test day, they had all recovered. In the current study, the effect of vitamin C was calculated as the difference in the log(PC_20_) levels between the baseline and 1 hour after vitamin C administration. A paired t-test was used to calculate the *P*-value and the 95% CI in the log-scale was also obtained. Thereafter the 95% CI was converted into the ratio scale. The paired t-test of the log(PC_20_) values was also used to calculate the interaction *P*-value between the vitamin C effect and the presence of the common cold. See Additional files 2 and 3 for the calculations.

**Figure 1:**
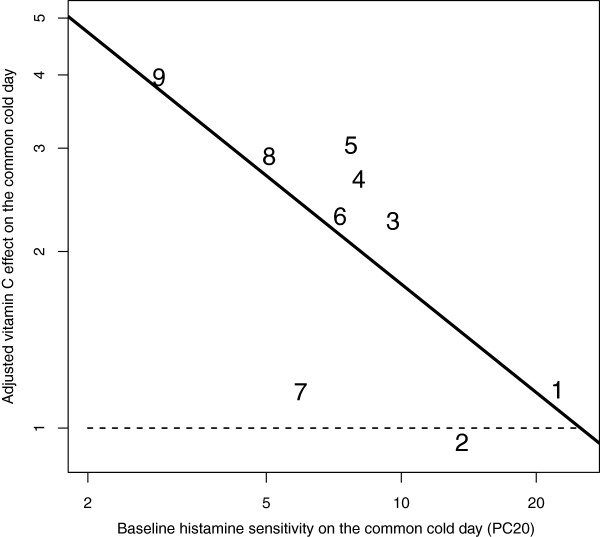
**The association between vitamin C effect and baseline histamine PC**_**20 **_**level on the common-cold-day.** Baseline PC_20_ level indicates the histamine PC_20_ level before vitamin C administration on the common-cold day in the Bucca et al. study [25,26]. Adjusted vitamin C effect indicates that the vitamin C effect of the second day (after recovery at 6 wk) is subtracted from the vitamin C effect on the common-cold-day. For example, participant #9 had a 7.45-fold increase in PC_20_ level on the common-cold-day and a 1.88-fold increase in PC_20_ level on the second day. This gives an adjusted vitamin C effect of 3.96 (7.45/1.88). Adding the baseline histamine PC_20_ level to the null model increased the model fit by χ^2^(1 df) = 6.2, P = .013. The horizontal dash (−) line indicates the level of vitamin C effect after recovery. The numbers indicate the identification numbers used in Additional file 2. See the Additional file 3 for the calculations of the linear model.

In the analysis of the Bucca et al. data, linear modeling (lm program of the R package [28]) was used to determine whether the effect of vitamin C on the common-cold-day could be explained by the baseline log(PC_20_) level values a) on the common-cold-day or b) on the day the participant had recovered, or c) by the vitamin C effect on the day the participant had recovered. The improvement of model fit was assessed by the likelihood ratio test. The vitamin C effect on the common-cold-day was significantly explained by the baseline log(PC_20_) level on the common-cold-day and by the vitamin C effect on the day the participant had recovered. Compared with the null model without the explanatory variables, the addition of these two variables improved the model fit by χ^2^(2 df) = 9.1, *P* = .011. Since the vitamin C effect on the common-cold-day was explained by the vitamin C effect on the day the participant had recovered, the latter was subtracted from the former, which gave an adjusted vitamin C effect. In a linear model, the adjusted vitamin C effect was significantly explained by the baseline log(PC_20_) level: χ^2^(1 df) = 6.2, *P* = .013 (Figure 1). See Additional file 3 for the calculations.

In the Bucca et al. data, the association between the histamine PC_20_ levels on the two study days separated by a 6 week period was analyzed using the “cor.test” and “lm” programs of the R package [28]. The influence of vitamin C administration on the association between the log(PC_20_) levels on the two study days was analyzed with linear modeling as follows. First, the baseline histamine log(PC_20_) determined when the participant was suffering from the common cold was modeled using the baseline log(PC_20_) determined after the participant had recovered as the explanatory variable. Second, the difference in the vitamin C effects on the two study days was added to the linear model. The comparison of these two models tested whether vitamin C administration significantly improved the association between the PC_20_ levels on the two study days separated by 6 weeks and gave χ^2^(1 df) = 9.2, *P* = .0024. See Additional file 3 for the calculations.

The Bucca et al. study did not use a placebo and the second histamine challenge test was carried out at 1 hour after the baseline test. Therefore, studies that give information on the role of placebo on the histamine challenge test [7-9,33-35] and about tachyphylaxis [35-40] were searched; see Additional file 2 for the data of two reports [33,36]. Furthermore, one day before the first vitamin C test day, Bucca et al. ascertained the reproducibility of the histamine challenge test. The baseline test and the test 1 hour later had a very close correlation (r = .96). The coefficient of variation was on average 6% for three measurements comprising the two reproducibility day measurements and the baseline histamine test on the first vitamin C day [25].

The 2-tailed *P*-values are presented in this text.

The statistical analyses in this systematic review were not planned in a protocol prior to the review. Instead the statistical approaches were formulated after the data of the selected studies became available.

## Results

Three intervention studies that give information on the effect of vitamin C on common cold-induced asthma were identified. A total of 79 people participated in the three trials (Table 1). The three studies are clinically heterogeneous and the outcomes are different. Therefore no pooled effect can be calculated. Instead the studies are analyzed separately. The methodological characteristics of the three studies are described in Table 2.

The study by Anah et al. was a randomized double-blind placebo-controlled trial with parallel groups (N = 41) [27]. The effect of 1 g/day of vitamin C on participants who had histories of increased asthma attacks during the rainy season in Nigeria was investigated. In all previous cases their attacks were precipitated by respiratory infections, which started with a sore throat and a dry cough. The 14-week trial was carried out during the Nigerian rainy season. The study recorded 35 asthma attacks in the placebo group (n = 19), but only 9 attacks in the vitamin C group (n = 22). Thus, vitamin C decreased the incidence of all asthma exacerbations by 78% (Table 3). The effect appeared even greater on those asthma exacerbations that were classified as severe or moderate, which decreased by 89% (Table 3). Furthermore, Anah et al. reported that there was a recurrence of asthma attacks in the vitamin C group within 8 weeks after vitamin administration was discontinued, though no quantitative data were published.

The study by Schertling et al. was a randomized double-blind placebo-controlled cross-over trial conducted in the former East Germany (N = 29) [30,31]. The effect of 5 g/day of vitamin C was studied on participants who had a diagnosis of infection-related asthma. Schertling et al. tested bronchial responsiveness to histamine so that hypersensitivity was defined as increase in respiratory tract resistance of 50% for a cumulative exposure to <1 μmol histamine. Vitamin C decreased the proportion of participants who were sensitive to histamine by 52 percentage points (Table 3). The decrease in prevalence was from 91% (21/23) during the placebo phase to 39% (9/23) during the vitamin C phase. The mean symptom scores and PEF values were also reported and, though non-significant, their differences were in favor of vitamin C (Table 3).

Bucca et al. investigated the effect of a single dose 1 g vitamin C on histamine challenge test of common cold patients in a self-controlled study (n = 9) [25,26]. A second pair of histamine challenge tests was carried out 6 weeks later after the participants had recovered. When the participants suffered from the common cold, the baseline PC_20_ level was 50% lower than after they had recovered (*P* = .005), which indicates that the common cold increased bronchial sensitivity to histamine. When the participants suffered from the common cold, vitamin C administration caused a 3.2-fold increase in the geometric mean histamine PC_20_ level in the baseline values of 7.8 to 25.1 mg/ml (Table 3). After the participants had recovered from the common cold 6 weeks later, vitamin C increased the PC_20_ level by just 1.6 fold.

A comparison between the two study days found that there was a significant interaction between the vitamin C effect and the presence of the common cold (*P* = .003), which indicates that the effect of vitamin C on bronchial hypersensitivity was different between the two test days separated by 6 weeks. Furthermore, a linear regression analysis revealed that the difference in the vitamin C effect between the two study days depended significantly on the baseline histamine PC_20_ level determined on the common-cold-day (Figure 1). If there are factors causing bias in the self-controlled comparison and if the factors are constant on both study days, they would be removed from the calculation of the adjusted vitamin C effect, i.e., the difference in effect between the two study days. Such potential factors include the placebo effect and tachyphylaxis. The linear regression model indicated there were no differences in the vitamin C effects between the two study days when the baseline histamine PC_20_ level was 25 mg/ml on the common-cold-day (Figure 1). However, when the baseline PC_20_ level was 2 mg/ml on the common-cold-day, the model predicted that vitamin C administration would increase the histamine PC_20_ level 4.7-fold over the corresponding effect after recovery from the cold.

In the study by Bucca et al., there was a significant correlation between the histamine PC_20_ levels on the two study days after vitamin C administration (r = 0.81, *P* = .008). After vitamin C administration the geometric means of the PC_20_ levels on the two days were essentially identical: 25.1 vs. 25.7 mg/ml [25]. Before vitamin C administration the correlation between the PC_20_ levels for the two days was weak (r = 0.66, *P* = .054). Linear modeling was used to determine whether the increase in correlation caused by vitamin C administration was statistically significant. Adding the difference between the vitamin C effects for the two study days as a factor to the linear model explaining the baseline PC_20_ levels on the common-cold-day by the baseline PC_20_ levels after recovery improved the fit of the linear model significantly (*P* = .003). Consequently, the closer association between the PC_20_ values after vitamin C administration cannot be explained by random variation alone.

Bucca et al. did not use a placebo [25], and therefore data on the possible role of placebo on the histamine challenge test was assessed from other studies. One study reported that the histamine sensitivity on the placebo day did not differ from the levels on the no-treatment day (95% CI: -22% to +21%) [33]. Other studies also found no effect of placebo on histamine sensitivity [7-9,34,35]. Another potential problem in the Bucca group’s study design was tachyphylaxis, which indicates that a second histamine challenge test carried out too soon after the first test might lead to increased PC_20_ values. Although this phenomenon has been reported, in one study the increase in histamine PC_20_ value was less than 1.5-fold for the second challenge test carried out at 1 hour after the first test [36]. Other studies have found small or no tachyphylaxis effects [35,37-40]. Furthermore, the close reproducibility of the histamine challenge test in the Bucca et al. study is also inconsistent with a substantial tachyphylaxis effect [25]. Finally, if there is a constant placebo effect or tachyphylaxis that would cause bias, such effects would be eliminated from the calculation of the adjusted vitamin C effect, i.e., the difference in effects between the two study days. Therefore, the strong association between the adjusted vitamin C effect and the baseline histamine PC_20_ level is a further argument against the placebo effect and the tachyphylaxis effect (Figure 1). In conclusion, the placebo effect is not an issue and tachyphylaxis does not explain the 3.2-fold increase in the histamine PC_20_ level of common cold patients who were administered vitamin C.

## Discussion

The three identified studies give relevant information for assessing the potential role of vitamin C on alleviating asthma exacerbations caused by the common cold. The studies differ substantially in their methods, participants, settings and outcomes, yet each of them found a benefit from vitamin C administration.

Anah et al. [27] recorded the occurrence of asthma exacerbations, whereas Schertling et al. [30] and Bucca et al. [25] studied bronchial sensitivity to histamine. The common cold can lead to a transient bronchial hypersensitivity, which is a characteristic feature of asthma [7-12,25]. Challenge tests with histamine and methacholine have been widely used for the examinations of asthma patients [41]. Furthermore, reducing the airway hypersensitivity of asthmatics led to a significant reduction in asthma exacerbations, which implies that bronchial hypersensitivity is a clinically important measure of the asthma severity [42].

Two of the identified studies [27,30] were randomized double-blind placebo-controlled trials. Both studies used patients who suffered from infection-related asthma. Anah et al. found that vitamin C decreased the occurrence of respiratory infection-induced asthma attacks by 78% [27]. Schertling et al. found that vitamin C decreased the proportion of asthma patients who suffered from bronchial hypersensitivity to histamine by 58 percentage points [30]. In the Schertling group’s study, vitamin C did not influence asthma symptoms or PEF values. However, the number of participants in that study was small and therefore the study had insufficient statistical power to test the effect on these outcomes. The Anah et al. study was carried out in Nigeria in the 1970s, and Schertling et al. study was carried out in former East Germany in the 1980s. Thus, those findings cannot be directly extrapolated to Western countries in the 2010s. Nevertheless, these two trials were methodologically strong. The highly significant effects caused by vitamin C administration indicate a genuine biological effect on the lungs of some people who suffer from common cold-induced asthma exacerbations.

Bucca et al. found that vitamin C administration caused a 3.2-fold increase in histamine PC_20_ levels of common cold patients, which indicates that vitamin C decreased bronchial hypersensitivity caused by the common cold [25]. The effect of vitamin C was significantly smaller after the participants had recovered from the colds. Furthermore, on the two vitamin C test days, which were separated by 6 weeks, histamine PC_20_ levels correlated significantly after vitamin C was administered, but did not do so before its administration. This indicates that vitamin C administration was associated with a kind of normalization of bronchial sensitivity. The study by Bucca and colleagues is methodologically weaker than the two other studies, but analyzing the two study days gives much strength compared with measuring participants only on the common-cold-day. In any case, placebo effect and tachyphylaxis do not readily explain the effect of vitamin C found in the participants when they were suffering from the common cold.

Publication bias might be a problem in the case where a few studies have been published. However, publication bias cannot reasonably explain the remarkably small *P*-values found in each of the three studies reviewed here. Furthermore, publication bias cannot explain findings that are not published in the original study reports. Therefore, publication bias cannot explain the association between the PC_20_ level on the common-cold day and the adjusted vitamin C effect (Figure 1). This systematic review was done by one person and one person might have a higher error rate in the extraction of data than a group. However, only three studies are included and the extracted data were several times compared against the original study reports. It is unlikely that errors would have remained. Furthermore, to increase transparency in this systematic review, the extracted data and the calculations are described in Additional files 2 and 3.

Asthma is a heterogeneous syndrome, an “umbrella concept,” that comprises a collection of different phenotypes with different underlying pathophysiologies, rather than a single disease [43,44]. A previous meta-analysis found that vitamin C may alleviate exercise-induced bronchoconstriction [24] and the current study revealed that vitamin C may alleviate common cold-induced asthma exacerbations. It is noteworthy that both of these conditions involve short-term stress, caused either by physical exertion or by an infectious disease. Given the diverse asthma phenotypes that exist, it is relevant to consider whether vitamin C might influence other asthma phenotypes.

In a four-month study of British asthmatics who regularly used inhaled corticosteroids, Fogarty et al. found no effect of 1 g/day vitamin C on the FEV_1_ level, on bronchial sensitivity to methacholine, or on asthma symptoms [45]. However, those authors found that the need for inhaled corticosteroids was slightly lower in the vitamin C group [46]. The Fogarty et al. study indicates that regular vitamin C administration is not substantially beneficial for patients with persistent asthma without acute problems. However, their study does not conflict with the possibility that vitamin C may be beneficial for pulmonary functions of some asthmatics under certain forms of acute stress, such as people who endure heavy physical activity or suffer from a viral respiratory tract infection.

Evidently, more research on the role of vitamin C on common cold-induced asthma is needed. On the other hand, vitamin C costs only a few pennies per gram and it is safe in gram doses [16,17,47]. Given the strong evidence that shows that vitamin C alleviates common cold symptoms [14-17], and the findings of this systematic review, it may be reasonable for asthmatic patients to test vitamin C on an individual basis when they have exacerbations of asthma caused by respiratory infections.

## Abbreviations

CI: Confidence interval; FEV1: Forced expiratory volume in 1 second; PC20: Concentration of histamine needed for a 20% FEV_1_ decrease; PEF: Peak expiratory flow; RR: Rate ratio.

## Competing interests

The author declares that he has no competing interests.

## Supplementary Material

Additional file 1 Flow diagram of the literature. Click here for file

Additional file 2 Extraction and analysis of data. Click here for file

Additional file 3 Analysis of data: R-program printouts. Click here for file
