# Supplementary material for: Vitamin C and common cold-induced asthma: a systematic review and statistical analysis
Source: Allergy Asthma Clin Immunol. 2013 Nov 26;9(1):46. doi: 10.1186/1710-1492-9-46 (PMC4018579; doi:10.1186/1710-1492-9-46)

Supplementary file 1

Vitamin C and common cold-induced asthma:
a systematic review and statistical analysis
Harri Hemilä

Flow diagram of the literature search September 2013

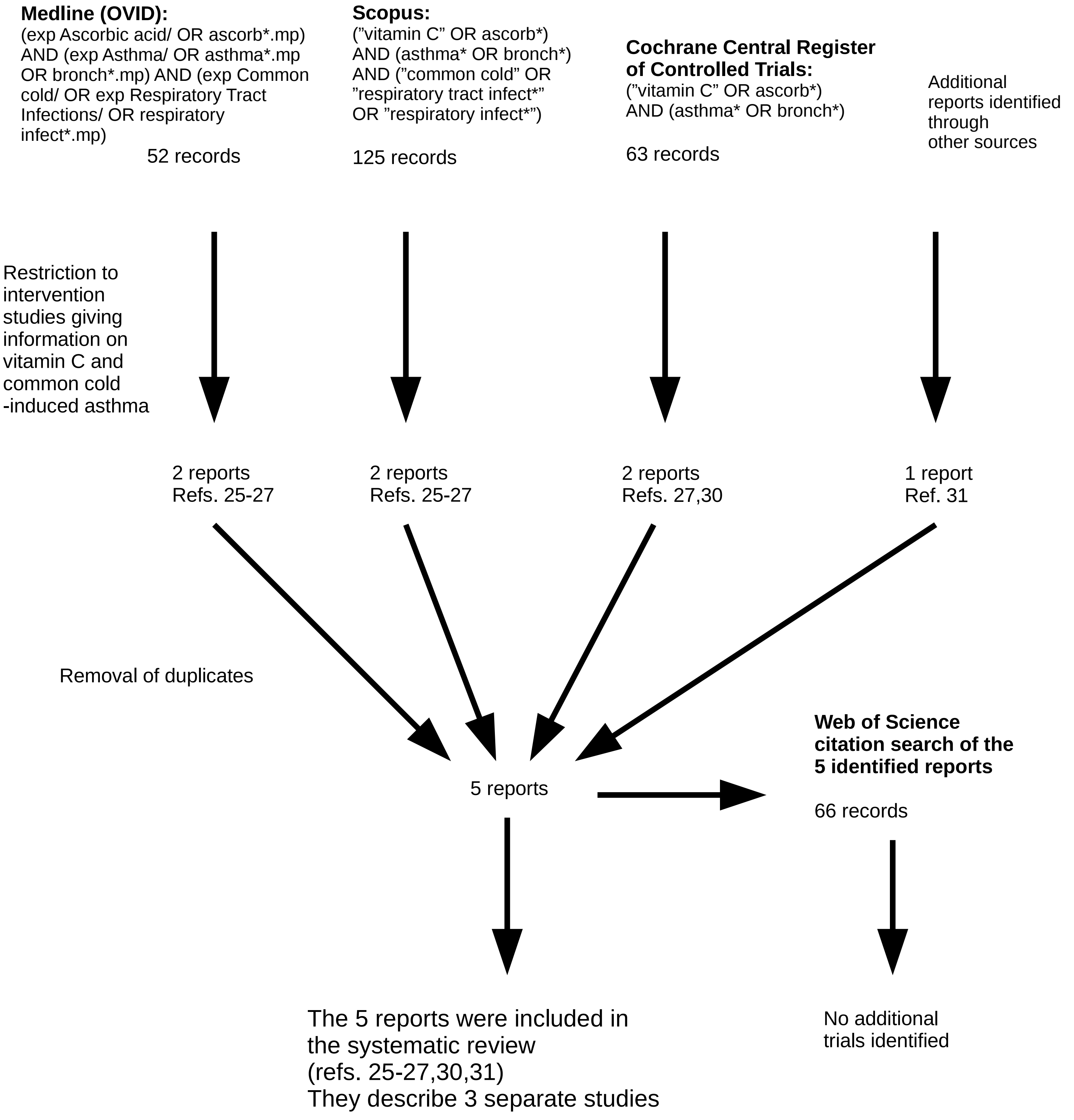

Supplement: Additional file 1 — Flow diagram of the literature. [file 1710-1492-9-46-S1.pdf]
