# Supplementary material for: Vitamin C and common cold-induced asthma: a systematic review and statistical analysis
Source: Allergy Asthma Clin Immunol. 2013 Nov 26;9(1):46. doi: 10.1186/1710-1492-9-46 (PMC4018579; doi:10.1186/1710-1492-9-46)
Supplement: Additional file 3 — Analysis of data: R-program printouts. [file 1710-1492-9-46-S3.pdf]

Supplementary file 3

Vitamin C and common cold-induced asthma:
a systematic review and statistical analysis

Submitted to: Allergy, Asthma & Clinical Immunology

R-program printouts (8 Oct 2013)

Harri Hemilä
Department of Public Health
University of Helsinki
Helsinki
Finland
harri.hemila@helsinki.fi
http://www.mv.helsinki.fi/home/hemila/
http://www.mv.helsinki.fi/home/hemila/vitc_asthma.htm (papers on vitC-asthma)

Contents

Page

2 **Anah 1980: imputed data set**
Anah data for the negative binomial model, see supplementary file 2

3 **Anah**
Poisson distribution confidence intervals

4 **Anah: all asthma attacks**
Negative binomial model confidence intervals

5 **Anah: severe and moderate asthma attacks**
Negative binomial model confidence intervals

6 **Bucca 1989: reconstructed data set**
Bucca data set for the statistical analysis
See supplementary file 2 for the construction of the data set
The data is the log(PC20) levels

7-9 **Bucca 1989: Bucca's AOV calculations and correct paired t-test**
In the 1989 paper, Bucca et al. used the analysis of variance (AOV) to calculate the effect of vitamin C and to compare the “During Cold” and “After Recovery” days. However, the data are paired (4 measurements with the same person) and therefore paired t-test is correct. AOV corresponds to un-paired t-test

7 Comparison of baseline (pre) vs. post vitamin C on the "During Cold” day
8 Comparison of baseline (pre) vs. post vitamin C on the "After Recovery” day
9 Comparison of baseline (pre) values for the “During Cold” and “After Recovery” days

10 Interaction test: difference in vitamin C effect on the "During Cold"
and "After Recovery” days

11 Correlations of pre and post vit C values between the two test days
12- Test of improvement of association when vitamin C effect is added to model
14- What can explain effect of vitamin C on the common cold day i.e. Cold_VC

```
> # Imputed Anah data set: Group 1 is vitamin C, Group 0 is Placebo

> Anah

   Participant Group Severe Moderate Mild SevMod All

1            1     1      1        0    2      1   3
2            2     1      1        0    0      1   1
3            3     1      0        1    0      1   1
4            4     1      0        0    4      0   4
5            5     1      0        0    0      0   0
6            6     1      0        0    0      0   0
7            7     1      0        0    0      0   0
8            8     1      0        0    0      0   0
9            9     1      0        0    0      0   0
10          10     1      0        0    0      0   0
11          11     1      0        0    0      0   0
12          12     1      0        0    0      0   0
13          13     1      0        0    0      0   0
14          14     1      0        0    0      0   0
15          15     1      0        0    0      0   0
16          16     1      0        0    0      0   0
17          17     1      0        0    0      0   0
18          18     1      0        0    0      0   0
19          19     1      0        0    0      0   0
20          20     1      0        0    0      0   0
21          21     1      0        0    0      0   0
22          22     1      0        0    0      0   0

23          23     0      3        0    0      3   3
24          24     0      3        0    0      3   3
25          25     0      2        0    0      2   2
26          26     0      0        4    1      4   5
27          27     0      0        4    1      4   5
28          28     0      0        4    1      4   5
29          29     0      0        2    2      2   4
30          30     0      0        2    2      2   4
31          31     0      0        0    4      0   4
32          32     0      0        0    0      0   0
33          33     0      0        0    0      0   0
34          34     0      0        0    0      0   0
35          35     0      0        0    0      0   0
36          36     0      0        0    0      0   0
37          37     0      0        0    0      0   0
38          38     0      0        0    0      0   0
39          39     0      0        0    0      0   0
40          40     0      0        0    0      0   0
41          41     0      0        0    0      0   0
```

> # All Anah asthma attacks ** "Poisson.Test" program

```
> poisson.test(c(9, 35), c(22, 19))

      Comparison of Poisson rates

data:  c(9, 35) time base: c(22, 19)
count1 = 9, expected count1 = 23.61, p-value = 1.122e-05
alternative hypothesis: true rate ratio is not equal to 1
95 percent confidence interval:
 0.09387772 0.47128869
sample estimates:
rate ratio
 0.2220779
```

> # Severe and moderate Anah asthma attacks ** "Poisson.Test" program

```
> poisson.test(c(3, 23), c(22, 19))

      Comparison of Poisson rates

data:  c(3, 23) time base: c(22, 19)
count1 = 3, expected count1 = 13.951, p-value = 1.15e-05
alternative hypothesis: true rate ratio is not equal to 1
95 percent confidence interval:
 0.02165246 0.37285085
sample estimates:
rate ratio
 0.1126482
```

> # All Anah asthma attacks ** negative binomial model

```
> Anah_All <- glm.nb(All~Group, data=Anah)

> summary(Anah_All)

Call:
glm.nb(formula = All ~ Group, data = Anah, init.theta = 0.3630726678,
    link = log)

Deviance Residuals:
    Min       1Q   Median       3Q      Max
-1.1445  -0.7402  -0.7402   0.2958   1.7691

Coefficients:
            Estimate Std. Error z value Pr(>|z|)
(Intercept)   0.6109     0.4166   1.467   0.1425
Group        -1.5047     0.6402  -2.350   0.0188 *
---
Signif. codes:  0 '***' 0.001 '**' 0.01 '*' 0.05 '.' 0.1 ' ' 1

(Dispersion parameter for Negative Binomial(0.3631) family taken to be 1)

    Null deviance: 36.522  on 40  degrees of freedom
Residual deviance: 30.907  on 39  degrees of freedom
AIC: 109.77

Number of Fisher Scoring iterations: 1


              Theta:  0.363
          Std. Err.:  0.179

 2 x log-likelihood:  -103.767

> qt(0.975, 40)
[1] 2.021075

> logRRnbAll<-c(-1.5047, -1.5047-2.021*0.6402, -1.5047+2.021*0.6402)

> logRRnbAll
[1] -1.5047000 -2.7985442 -0.2108558

> exp(logRRnbAll)
[1] 0.22208391 0.06089865 0.80989084   # 95% CI is in bold
```

```
> # Severe and moderate Anah asthma attacks ** negative binomial model

> Anah_SevMod <- glm.nb(SevMod~Group, data=Anah)

> summary(Anah_SevMod)

Call:
glm.nb(formula = SevMod ~ Group, data = Anah, init.theta = 0.6089002017,
    link = log)

Deviance Residuals:
    Min       1Q   Median       3Q      Max
-1.1695  -1.1695  -0.4961   0.3262   1.2282

Coefficients:
            Estimate Std. Error z value Pr(>|z|)
(Intercept)   0.2336     0.3579   0.653  0.51394
Group        -2.2260     0.7322  -3.040  0.00236 **
---
Signif. codes:  0 '***' 0.001 '**' 0.01 '*' 0.05 '.' 0.1 ' ' 1

(Dispersion parameter for Negative Binomial(0.6089) family taken to be 1)

    Null deviance: 39.703  on 40  degrees of freedom
Residual deviance: 28.221  on 39  degrees of freedom
AIC: 82.886

Number of Fisher Scoring iterations: 1


              Theta:  0.609
          Std. Err.:  0.406

 2 x log-likelihood:  -76.886

> logRRnbSevMod<-c(-2.2260, -2.2260-2.021*0.7322, -2.2260+2.021*0.7322)

> logRRnbSevMod
[1] -2.2260000 -3.7057762 -0.7462238

> exp(logRRnbSevMod)
[1] 0.10795941 0.02458113 0.47415368   # 95%CI is in bold
```

Histamine Log(PC20) measurements:

These four variables are measured from Bucca (1992) fig.1 (suppl file 2)
Cold_pre: Participants **had** the common cold, level **before** vitamin C
Cold_post: Participants **had** the common cold, level **after** vitamin C
Reco_pre: Participants had **recovered** of the cold, level **before** vitamin C
Reco_post: Participants had **recovered** of the cold, level **after** vitamin C

These variables are calculated from the four above

```
Cold_VC = Cold_post  - Cold_pre     (effect of vitamin C on common cold day)
Reco_VC = Reco_post  - Reco_pre     (effect of vitamin C on after recovery day)
Diff_VC     = Cold_VC - Reco_VC     (for analyzing the association on p.12)
Reco_pre_VC = Reco_pre - Diff_VC    (for analyzing the association on p.12)
Diff_VC_10  = 10exp(Diff_VC)        (vit C effect on the absolute scale)
Cold_pre_10 = 10exp(Cold_pre)       (baseline PC20 on the absolute scale)
```

```
> # reconstructed Bucca data set

> Bucca
  Person Cold_pre Cold_pre_10 Cold_post Cold_VC Reco_pre Reco_post Reco_VC Reco_pre_VC Diff_VC Diff_VC_10
1      1   1.3480     22.2831    1.6971  0.3491   1.6682    1.9520  0.2838      1.6029  0.0652     1.1621
2      2   1.1355     13.6600    1.4146  0.2792   1.0926    1.3960  0.3034      1.1168 -0.0242     0.9457
3      3   0.9812      9.5760    1.9114  0.9303   1.1024    1.6798  0.5775      0.7495  0.3528     2.2533
4      4   0.9052      8.0392    1.5554  0.6502   1.6756    1.9012  0.2256      1.2510  0.4246     2.6582
5      5   0.8880      7.7263    1.2706  0.3826   1.2021    1.1024 -0.0997      0.7197  0.4824     3.0365
6      6   0.8633      7.2991    1.1653  0.3020   1.0860    1.0269 -0.0592      0.7248  0.3612     2.2972
7      7   0.7756      5.9655    1.0800  0.3043   0.9504    1.1928  0.2424      0.8884  0.0620     1.1534
8      8   0.7053      5.0731    1.2301  0.5248   1.1406    1.2021  0.0615      0.6773  0.4633     2.9058
9      9   0.4597      2.8817    1.3317  0.8720   0.8292    1.1033  0.2740      0.2313  0.5980     3.9624
```

Data for Bucca AOV calculations
Cold: pre vitamin C and post vitamin C measurements on "Common Cold" day
Reco: pre vitamin C and post vitamin C measurements "After Recovery" day
Group: measurement pre or post vitamin C administration

Pre: pre vit C measurements on "During Cold" and "After Recovery" (6 week) days
GroupPre: day of the pre measurement ("During Cold" or "After Recovery")

> # Bucca data set for AOV calculations

```
> Bucca_aov
     Cold     Group   Reco    Pre GroupPre
1  1.3480  pre_vitC 1.6682 1.3480     Cold
2  1.1355  pre_vitC 1.0926 1.1355     Cold
3  0.9812  pre_vitC 1.1024 0.9812     Cold
4  0.9052  pre_vitC 1.6756 0.9052     Cold
5  0.8880  pre_vitC 1.2021 0.8880     Cold
6  0.8633  pre_vitC 1.0860 0.8633     Cold
7  0.7756  pre_vitC 0.9504 0.7756     Cold
8  0.7053  pre_vitC 1.1406 0.7053     Cold
9  0.4597  pre_vitC 0.8292 0.4597     Cold
10 1.6971 post_vitC 1.9520 1.6682     Reco
11 1.4146 post_vitC 1.3960 1.0926     Reco
12 1.9114 post_vitC 1.6798 1.1024     Reco
13 1.5554 post_vitC 1.9012 1.6756     Reco
14 1.2706 post_vitC 1.1024 1.2021     Reco
15 1.1653 post_vitC 1.0269 1.0860     Reco
16 1.0800 post_vitC 1.1928 0.9504     Reco
17 1.2301 post_vitC 1.2021 1.1406     Reco
18 1.3317 post_vitC 1.1033 0.8292     Reco
```

```
> # Bucca (1989) used Analysis of Variance [AOV] (corresponds to un-paired t-
test)
> # The appropriate comparison is by the paired t-test
> # The Bucca statistics are re-calculated to show that the reconstructed data
set gives the same F-statistics and to show that the Bucca calculations were un-
paired (should have been paired)

> # 1: AOV Comparison of pre vs. post vitamin C on the "During Cold" day

> aov.1 = aov(Cold~Group, data=Bucca_aov)

> summary(aov.1)
            Df Sum Sq Mean Sq F value   Pr(>F)
Group        1  1.173  1.1727   17.17 0.000764 ***
Residuals   16  1.093  0.0683

> # Bucca (1989) reported F = 17 which is consistent with the F value above

> # 1: UN-PAIRED t-test (corresponds to AOV)

> t.test(Bucca$Cold_post, Bucca$Cold_pre, alternative='two.sided',
conf.level=.95, paired=FALSE)

      Welch Two Sample t-test

data:  Bucca$Cold_post and Bucca$Cold_pre
t = 4.1434, df = 15.934, p-value = 0.00077
alternative hypothesis: true difference in means is not equal to 0
95 percent confidence interval:
 0.2492174 0.7717604
sample estimates:
mean of x mean of y
1.4062444 0.8957556

> # F = t*t  >  17.1 = 4.14*4.14; AOV and un-paired t-test give the same result



> # 1: PAIRED t-test  (the correct test for paired data)

> t.test(Bucca$Cold_post, Bucca$Cold_pre, alternative='two.sided',
conf.level=.95, paired=TRUE)

      Paired t-test

data:  Bucca$Cold_post and Bucca$Cold_pre
t = 6.0747, df = 8, p-value = 0.0002977
alternative hypothesis: true difference in means is not equal to 0
95 percent confidence interval:
 0.3167040 0.7042738
sample estimates:
mean of the differences
              0.5104889

> # 95% CI to absolute PC20 values for the "Common Cold day"
> log_PC20<-c(0.3167, 0.7042738, 0.5104889)
> log_PC20
[1] 0.3167000 0.7042738 0.5104889
> PC20<-10^(log_PC20)
> PC20
[1] 2.073481 5.061437 3.239581
```

```
> # 2: AOV of pre vs. post vit C on the "After Recovery" day (6 wk later)

> aov.2 = aov(Reco~Group, data=Bucca_aov)

> summary(aov.2)
            Df Sum Sq Mean Sq F value Pr(>F)
Group        1 0.1819  0.1819   1.698  0.211
Residuals   16 1.7144  0.1071

> # Bucca (1989) reported F = 1.7 which is consistent with the F value above

> # 2: UN-PAIRED t-test (corresponds to AOV)

> t.test(Bucca$Reco_post, Bucca$Reco_pre, alternative='two.sided',
conf.level=.95, paired=FALSE)

      Welch Two Sample t-test

data:  Bucca$Reco_post and Bucca$Reco_pre
t = 1.3029, df = 15.362, p-value = 0.2118
alternative hypothesis: true difference in means is not equal to 0
95 percent confidence interval:
 -0.1271803  0.5292692
sample estimates:
mean of x mean of y
 1.395167  1.194122

> # F = t*t  >  1.7 = 1.3*1.3; AOV and un-paired t-test give the same result



> # 2: PAIRED t-test (the correct test for paired data)

> t.test(Bucca$Reco_post, Bucca$Reco_pre, alternative='two.sided',
conf.level=.95, paired=TRUE)

      Paired t-test

data:  Bucca$Reco_post and Bucca$Reco_pre
t = 2.9077, df = 8, p-value = 0.01966
alternative hypothesis: true difference in means is not equal to 0
95 percent confidence interval:
 0.04160047 0.36048842
sample estimates:
mean of the differences
              0.2010444

> # 95% CI to absolute PC20 values for the "After Recovery day"
> log_PC20_reco<-c(0.04163681, 0.36056319, 0.2011)
> log_PC20_reco
[1] 0.04163681 0.36056319 0.20110000
> PC20_reco<-10^(log_PC20_reco)
> PC20_reco
[1] 1.100619 2.293840 1.588913

> # Bucca (1989) reports that for the "being recovered" day, vitamin C effect
P>0.05
> # that is based on the AOV, whereas the paired t-test gives P=0.02 (above)
```

```
> # 3: AOV of pre vit C PC20 values on the "During Cold" and "After Recovery"
days

> aov.3 = aov(Pre~GroupPre, data=Bucca_aov)

> summary(aov.3)
            Df Sum Sq Mean Sq F value Pr(>F)
GroupPre     1 0.4006  0.4006   5.369 0.0341 *
Residuals   16 1.1938  0.0746

> # Bucca (1989) reported F = 5.23 which is consistent with the F value above

> # 3: UN-PAIRED t-test (corresponds to AOV)

> t.test(Bucca$Cold_pre, Bucca$Reco_pre, alternative='two.sided',
conf.level=.95, paired=FALSE)

      Welch Two Sample t-test

data:  Bucca$Cold_pre and Bucca$Reco_pre
t = -2.3171, df = 15.678, p-value = 0.03438
alternative hypothesis: true difference in means is not equal to 0
95 percent confidence interval:
 -0.57179828 -0.02493506
sample estimates:
mean of x mean of y
0.8957556 1.1941222


> # F = t*t  >  5.38 = 2.32*2.32; AOV and un-paired t-test give the same result

> # 3: PAIRED t-test (the correct test for paired data)

> t.test(Bucca$Cold_pre, Bucca$Reco_pre, alternative='two.sided',
conf.level=.95, paired=TRUE)

      Paired t-test

data:  Bucca$Cold_pre and Bucca$Reco_pre
t = -3.9244, df = 8, p-value = 0.004391
alternative hypothesis: true difference in means is not equal to 0
95 percent confidence interval:
 -0.4736911 -0.1230422
sample estimates:
mean of the differences
             -0.2983667

> log_PC20_pre_diff<-c(-0.4736911, -0.1230422, -0.2983667)
> log_PC20_pre_diff
[1] -0.4736911 -0.1230422 -0.2983667
> PC20_pre_diff<-10^(log_PC20_pre_diff)
> PC20_pre_diff
[1] 0.3359765 0.7532824 0.5030757
```

> # Interaction test: difference in the vitamin C effect between the "During Cold" and "After Recovery" days

```
> # Small P-value means that the effect of vitamin C on the two study days is different
```

> t.test(Bucca$Cold_VC, Bucca$Reco_VC, alternative='two.sided', conf.level=.95, paired=TRUE)

```
      Paired t-test

data:  Bucca$Cold_VC and Bucca$Reco_VC
t = 4.222, df = 8, p-value = 0.002908
alternative hypothesis: true difference in means is not equal to 0
95 percent confidence interval:
 0.1404389 0.4784944
sample estimates:
mean of the differences
              0.3094667
```

> #the t-test above is identical with the linear model:

> LinearModel.1 <- lm(Cold_VC - Reco_VC ~ 1, data=Bucca)

```
> summary(LinearModel.1)

Call:
lm(formula = Cold_VC - Reco_VC ~ 1, data = Bucca)

Residuals:
     Min       1Q   Median       3Q      Max 
-0.33367 -0.24417  0.05173  0.15383  0.28853 

Coefficients:
            Estimate Std. Error t value Pr(>|t|)   
(Intercept)   0.3095     0.0733   4.222  0.00291 **

Residual standard error: 0.2199 on 8 degrees of freedom
```

> # and this is identical with (since Diff_VC = Cold_VC - Reco_VC, see p. 6)

> LinearModel.2 <- lm(Diff_VC ~ 1, data=Bucca)

```
> summary(LinearModel.2)

Call:
lm(formula = Diff_VC ~ 1, data = Bucca)

Residuals:
     Min       1Q   Median       3Q      Max 
-0.33368 -0.24428  0.05172  0.15382  0.28852 

Coefficients:
            Estimate Std. Error t value Pr(>|t|)   
(Intercept)   0.3095     0.0733   4.222  0.00291 **

Residual standard error: 0.2199 on 8 degrees of freedom
```

Models 1 and 2 are the basis for a further model on pages 15 and 16

> # Correlation of PC20 values BEFORE vit C on the "During Cold" and "After Recovery" days

```
> cor.test(Bucca$Cold_pre,  Bucca$Reco_pre,  alternative="two.sided",
method="pearson")

      Pearson's product-moment correlation

data:  Bucca$Cold_pre and Bucca$Reco_pre
t = 2.313, df = 7, p-value = 0.05395
alternative hypothesis: true correlation is not equal to 0
95 percent confidence interval:
 -0.01056197  0.92010969
sample estimates:
      cor
0.6581765
```

> # Correlation of PC20 values AFTER vit C on the "During Cold" and "After Recovery" days

```
> cor.test(Bucca$Cold_post, Bucca$Reco_post, alternative="two.sided",
method="pearson")

      Pearson's product-moment correlation

data:  Bucca$Cold_post and Bucca$Reco_post
t = 3.7229, df = 7, p-value = 0.007428
alternative hypothesis: true correlation is not equal to 0
95 percent confidence interval:
 0.3292318 0.9597136
sample estimates:
      cor
0.8151262
```

Since (see p. 6)
Cold_post = [Cold_pre + Cold_VC]
Reco_post = [Reco_pre + Reco_VC]

This indicates that the correlation after vitamin C
Cold_post <> Bucca$Reco_post
is identical with
[Cold_pre + Cold_VC] <> [Reco_pre + Reco_VC]

Since both sides have the vitamin C effect, they can be combined to the difference [Cold_VC - Reco_VC] = Diff_VC
Thus, the above correlation is related to the correlation

Cold_pre <> Reco_pre - Diff_VC

This correlation can be tested with linear regression

Let variable (p. 6)
Reco_pre_VC = [Reco_pre – (Cold_VC - Reco_VC)] = [Reco_pre - Diff_VC]

> # Correlation of log(PC20) values BEFORE vit C on the "During Cold" and Reco_pre_VC

```
> cor.test(Bucca$Cold_pre, Bucca$Reco_pre_VC, alternative="two.sided",
method="pearson")

      Pearson's product-moment correlation

data:  Bucca$Cold_pre and Bucca$Reco_pre_VC
t = 4.9229, df = 7, p-value = 0.001708
alternative hypothesis: true correlation is not equal to 0
95 percent confidence interval:
 0.5222220 0.9747504
sample estimates:
      cor
0.8808487
```

Thus, PC20 level before vitamin C administration on the "During Cold" day can be explained by the PC20 level before vitamin C on the "After Recovery" day and the difference in the vitamin C effects on the two days.
This approach allows testing the influence of vitamin C on the association between the PC20 levels on the two days ("During Cold" and "After Recovery").

On the next page, models 11 and 12 test whether the addition of Diff_VC to the linear model which has the Reco_pre variable improves the fit.

> # Below, the baseline log(PC20) level on the "During Cold" day is explained by the baseline log(PC20) level on the "After Recovery" day

```
> LinearModel.11 <- lm(Bucca$Cold_pre ~ 1 + Bucca$Reco_pre  , data=Bucca)
> summary(LinearModel.11)

Call:
lm(formula = Bucca$Cold_pre ~ 1 + Bucca$Reco_pre, data = Bucca)

Residuals:
    Min      1Q  Median      3Q     Max 
-0.2649 -0.1600  0.0187  0.1377  0.2976 

Coefficients:
               Estimate Std. Error t value Pr(>|t|)  
(Intercept)      0.2154     0.3018   0.714   0.4985  
Bucca$Reco_pre   0.5697     0.2463   2.313   0.0539 .

Residual standard error: 0.2035 on 7 degrees of freedom
Multiple R-squared: 0.4332,  Adjusted R-squared: 0.3522 
F-statistic:  5.35 on 1 and 7 DF,  p-value: 0.05395 
```

> # Below, the difference in vitamin C effects is added to model 1 above

```
> LinearModel.12 <- lm(Bucca$Cold_pre ~ 1 + Bucca$Reco_pre + Bucca$Diff_VC , data=Bucca)
> summary(LinearModel.12)

Call:
lm(formula = Bucca$Cold_pre ~ 1 + Bucca$Reco_pre + Bucca$Diff_VC, 
    data = Bucca)

Residuals:
     Min       1Q   Median       3Q      Max 
-0.17733 -0.05837  0.05335  0.05571  0.15985 

Coefficients:
               Estimate Std. Error t value Pr(>|t|)  
(Intercept)      0.5411     0.2195   2.465   0.0488 *
Bucca$Reco_pre   0.4792     0.1620   2.959   0.0253 *
Bucca$Diff_VC   -0.7030     0.2151  -3.268   0.0171 *

Residual standard error: 0.1318 on 6 degrees of freedom
Multiple R-squared: 0.7961,  Adjusted R-squared: 0.7282 
F-statistic: 11.71 on 2 and 6 DF,  p-value: 0.008476 
```

> # likelihood ratio test of the models 11 and 12 above
> # see rationalization: http://en.wikipedia.org/wiki/Likelihood-ratio_test
> # small P-value means that the added variable improves the association

> lrtest(LinearModel.11,LinearModel.12)
Likelihood ratio test

```
Model 1: Bucca$Cold_pre ~ 1 + Bucca$Reco_pre
Model 2: Bucca$Cold_pre ~ 1 + Bucca$Reco_pre + Bucca$Diff_VC
  #Df LogLik Df Chisq Pr(>Chisq)   
1   3  2.690                        
```
2   4 7.291 1 9.202   0.002418 **

```
> #   What can explain the effect of vitamin C on the common cold day.
i.e. the variable Cold_VC ?

> LinearModel.21 <- lm( Bucca$Cold_VC ~ 1 + Bucca$Cold_pre + Bucca$Reco_pre +
Bucca$Reco_VC , data=Bucca)
> summary(LinearModel.21)

Call:
lm(formula = Bucca$Cold_VC ~ 1 + Bucca$Cold_pre + Bucca$Reco_pre +
    Bucca$Reco_VC, data = Bucca)

Coefficients:
               Estimate Std. Error t value Pr(>|t|)
(Intercept)      0.6698     0.2533   2.645   0.0457 *
Bucca$Cold_pre  -0.8842     0.3171  -2.788   0.0385 *
Bucca$Reco_pre   0.3754     0.2674   1.404   0.2193
Bucca$Reco_VC    0.9176     0.2909   3.154   0.0253 *

Residual standard error: 0.1631 on 5 degrees of freedom
Multiple R-squared: 0.7385,   Adjusted R-squared: 0.5816
F-statistic: 4.707 on 3 and 5 DF,  p-value: 0.0642


> LinearModel.22 <- lm( Bucca$Cold_VC ~ 1 + Bucca$Cold_pre + Bucca$Reco_VC ,
data=Bucca)
> summary(LinearModel.22)

Call:
lm(formula = Bucca$Cold_VC ~ 1 + Bucca$Cold_pre + Bucca$Reco_VC,
    data = Bucca)

Coefficients:
               Estimate Std. Error t value Pr(>|t|)
(Intercept)      0.8652     0.2281   3.793  0.00904 **
Bucca$Cold_pre  -0.5840     0.2524  -2.314  0.05995 .
Bucca$Reco_VC    0.8377     0.3075   2.724  0.03446 *

Residual standard error: 0.1758 on 6 degrees of freedom
Multiple R-squared: 0.6354,   Adjusted R-squared: 0.5139
F-statistic: 5.229 on 2 and 6 DF,  p-value: 0.04846


> lrtest(LinearModel.21,LinearModel.22)
Likelihood ratio test

Model 1: Bucca$Cold_VC ~ 1 + Bucca$Cold_pre + Bucca$Reco_pre + Bucca$Reco_VC
Model 2: Bucca$Cold_VC ~ 1 + Bucca$Cold_pre + Bucca$Reco_VC
  #Df LogLik Df  Chisq Pr(>Chisq)
1   5 6.1962
2   4 4.7006 -1 2.9913    0.08371 .

> # thus, Reco_pre (baseline PC20 on the "after recovery" day) is unimportant
```

```
> LinearModel.23 <- lm( Bucca$Cold_VC ~ 1  , data=Bucca)

> summary(LinearModel.23)

Call:
lm(formula = Bucca$Cold_VC ~ 1, data = Bucca)

Coefficients:
            Estimate Std. Error t value Pr(>|t|)
(Intercept)  0.51050    0.08404   6.074 0.000298 ***

Residual standard error: 0.2521 on 8 degrees of freedom


> lrtest(LinearModel.22,LinearModel.23)
Likelihood ratio test

Model 1: Bucca$Cold_VC ~ 1 + Bucca$Cold_pre + Bucca$Reco_VC
Model 2: Bucca$Cold_VC ~ 1
  #Df LogLik Df Chisq Pr(>Chisq)
1   4 4.7006
2   2 0.1601 -2 9.081    0.01067 *

> # thus, baseline PC20 on the "common cold day" and the vitamin C effect on the "after recovery day" explain the vitamin C effect on the "common cold day"
```

> # when the vitamin C effects are combined to the difference Diff_VC we get the following models 31 and 32:

```
> LinearModel.31 <- lm(Bucca$Diff_VC ~ 1  , data=Bucca)

> summary(LinearModel.31)

Call:
lm(formula = Bucca$Diff_VC ~ 1, data = Bucca)

Coefficients:
            Estimate Std. Error t value Pr(>|t|)
(Intercept)   0.3095     0.0733   4.222  0.00291 **

Residual standard error: 0.2199 on 8 degrees of freedom
```

> # LinearModel.31 is identical with LinearModel.1 above,
> # because Diff_VC = Cold_VC - Reco_VC
> # thus 31 is the test of interaction between the two days

> #addition of the baseline histamine sensitivity on the common cold day

> LinearModel.32 <- lm(Bucca$Diff_VC ~ 1 + Bucca$Cold_pre , data=Bucca)

```
> summary(LinearModel.32)

Call:
lm(formula = Bucca$Diff_VC ~ 1 + Bucca$Cold_pre, data = Bucca)

Coefficients:
               Estimate Std. Error t value Pr(>|t|)
(Intercept)      0.8596     0.2158   3.984   0.0053 **
Bucca$Cold_pre  -0.6142     0.2328  -2.638   0.0335 *

Residual standard error: 0.1665 on 7 degrees of freedom
Multiple R-squared: 0.4986,   Adjusted R-squared: 0.427
F-statistic: 6.961 on 1 and 7 DF,  p-value: 0.0335
```

> lrtest(LinearModel.31,LinearModel.32)

```
Likelihood ratio test

Model 1: Bucca$Diff_VC ~ 1
Model 2: Bucca$Diff_VC ~ 1 + Bucca$Cold_pre
  #Df LogLik Df  Chisq Pr(>Chisq)
1   2 1.3905
2   3 4.4973  1 6.2135    0.01268 *
```

> # thus baseline histamine PC20 significantly improves the fit of the model
